# Supplementary material for: Patterns of Intron Gain and Loss in Fungi
Source: PLoS Biol. 2004 Nov 30;2(12):e422. doi: 10.1371/journal.pbio.0020422 (PMC532390; doi:10.1371/journal.pbio.0020422)
Supplement: Table S1 — Also available at http://genes.mit.edu/NielsenEtAl/. (4.3 MB ZIP). [file pbio.0020422.st001.zip › NielsenEtAl/html/384.html]

AN6364.1.NCU07554.1.MG04988.1.FG06754.1


```
 CLUSTAL W (1.82) Multiple Sequence Alignments - Introns Inserted


Sequence 1: NCU07554.1	1192 aa
Sequence 2: MG04988.1	1204 aa
Sequence 3: FG06754.1	1202 aa
Sequence 4: AN6364.1	1215 aa
Alignment Length: 1229 aa
Number Identitical Residues: 471 aa
Alignment Score (without introns) 24341


MG04988.1 	MYIKQIIIQGFKS2YKEQTVIEPFSPGTNVIVGRNGSGKSNFFAAIRFVLSDAYTQMSRE
NCU07554.1	MHIKQIIIQGFKS2YKEQTVIEPFSPGTNVIVGRNGSGKSNFFAAIRFVLSDAYTNMSRE
FG06754.1 	MYIKQIIIQGFKS2YKDQTVIEPFSSKTNVIVGRNGSGKSNFFAAIRFVLSDAYTQMSRE
AN6364.1  	MYVKQIIIQGFKS2YKDQTVIEPFSPKHNVIVGRNGSGKSNFFAAIRFVLSDAYTHLGRE
          	*::********** **:********.  ***************************::.**

MG04988.1 	ERQALLH~EGSGSAVMTAYVEVIFDNSDDRFHTGNKEVSIKRTISLKKDEYSIDKKVVQS
NCU07554.1	ERQALLH~EGSGSAVMSAYVEIIFDNTDKRFSEPGDEVVIRRTIGLKKDEYSVDKK-VQT
FG06754.1 	ERQGLLH~EGSGSAVMSAYVEIIFDNSDDRFPTGNKEVILRRTIGLKKDEYSVDRK-VVT
AN6364.1  	ERQALLH0EGSGSAVMSAYVEIIFDNSDERFPTGKPELVLRRTIGLKKDEYTLDRK-NAT
          	***.*** ********:****:****:*.**     *: ::***.******::*:*   :

MG04988.1 	KNEVFSMLEMAGFSRVNPYYIVPQGRVTALTNMKEADRLKLLKEIAGTESYEARRTESRK
NCU07554.1	RADVLKILETAGFAKENPFYIVPQGRVAAITNMKENERLNLLKEIAGTNLYDDRRIQSLK
FG06754.1 	KADVMNLLEAAGFSRSNPYYIVPQGRVTALTNMKESDRLNLLKEVAGTQVYETRRAESLK
AN6364.1  	KNDVMNLLESAGFSRSNPYYIVPQGRVTALTNMKDSERLNLLKEVAGTQVYEARRAESLK
          	: :*:.:** ***:: **:********:*:****: :**:****:***: *: ** :* *

MG04988.1 	IMHETINKREKIDELLKDVKERLQELEEEKEELRGFQAKDRERRCLEYAYYHHHQQVLTE
NCU07554.1	IMAETNSKREKIDELLEYIKERLSELEEEKDELRDFQEKDRERRCLEYAHWHRLQETNNN
FG06754.1 	IMNETNNKREKIDELLVYIKERLNELEEEKEELRGFQDKDRERRCLEYAYYHNIQLNVQS
AN6364.1  	IMHETNSKREKIDELLDFINERLAELEEEKDELRNFQEKDKERRCLEYTIYSREQQEIAS
          	** ** .*********  ::*** ******:***.** **:*******: : . *    .

MG04988.1 	ALEEIEQARLS-ADGNSEYQTRFAEGEKSIAQLESTLEELQQSLEMLRIDRQQLENDRRA
NCU07554.1	TLEQIEEVRQGGAGATTKDRVQLQKTEKEIAALDHKFQELKQTLELLAIERRQLDEDRKD
FG06754.1 	ALEELDNARQDGIDSSDTNRAEFSQGEKAISRLDSEIHKLEREMELLQIDRRQLEEDRRD
AN6364.1  	FLDSLEEQRQTGVEDTDINRDRFIQGEKEMAQVDAEIAECKQQIEFLKVDKAQLEDERRE
          	 *:.::: *  .   .   : .: : ** :: ::  : : :: :*:* ::: **:::*: 

MG04988.1 	TSKALAKVELKVKDLEQAQSTQTQSKKQHDAELKNVRAEIKTKEAELATVIPEYNKRKQK
NCU07554.1	TARSQAKAELKLKDLDETRHSREKAQQQQEAELNEVRRRIQAAESELAKITPDYEKWKKE
FG06754.1 	SAKSMAKAEMKTKNLKEGQSAQEQARAQHAADLESVQNEIASKEQQLSVILPAYNEKKQE
AN6364.1  	ASKALAQVELQAKSLSDNQAAAQESKARHDESLKAVQSAIEERQTELKELVPRFISAKDA
          	:::: *:.*:: *.*.: : :  ::: ::  .*: *:  *   : :*  : * : . *. 

MG04988.1 	EDAIRQQYDSAEATRQRLLDKQSRGARFKNKAERDKFLRREIQELGTSLNALKANRMELQ
NCU07554.1	EEEVAAARDLAATGRTRLLTKQTRSSQFRTKAERDAFLKSEIEETTMQLANQRANEMDAK
FG06754.1 	EDNVRRQLDHAESTRNRLFAKQSRGSQFRNKSERDAWLKKEIQELELNISTQKANKIDAD
AN6364.1  	EDAARAKLTEAETARQRLYAKQGRNSRFKNKSERDKWLQAEIKNNNASISSVQSVLSQTQ
          	*:        * : * **  ** *.::*:.*:*** :*: **::   .:   ::   : .

MG04988.1 	ESTVSTAIAIQTLEAEIAQLQQDLQTRGTRKEALNKQLMDAMNKENAMQDEK--------
NCU07554.1	EQVTLVENSIKQLEKEIQNIRDRLEGYGEDRSSIAEKLTKAQEAREHLYEER--------
FG06754.1 	EEVQRVHESIAQAEQEVAELRNRLANFSAERGALEEEATKAGDVIDKLNDER--------
AN6364.1  	EDINDIENDIALLEPETERLRQQIDGRGDTIQSVEQQVQAAKDERDRLMDQRKYVDWPRT
          	*.       *   * *  .::: :   .    :: ::   * :  : : :::.  . . :

MG04988.1 	--------LELRREADKLEARIATARRQREKADHRLGQAMDSATSRALDKIARLKRERNI
NCU07554.1	--------KRLRREEDKLSSLLANTRADRDNAEADLSHAMDSATARGLAAIRRLKRERDI
FG06754.1 	--------KLVRREDDKLNSVIANARQEKEAAERELSHTMDGATARGLATIRRLKQERDI
AN6364.1  	SCATLTIHRELWREEAKLDSILINASNEVDRAERNLSQMMDHNTSRGIAAVRRIKRQHNL
          	:.:: :    : **  **.: : .:  : : *:  *.: **  *:*.:  : *:*:::::

MG04988.1 	PGAYGILADLMQVD-DIYRVPVEQVAGNSLFHYVVDNKETGTMILDLLEKEKSGRVTLMP
NCU07554.1	PGAYGTLAELMNVPVDAYRLPVEQVAGNSLFHYVVDNEKTGTMLSEYLHKNYGGRVTFMP
FG06754.1 	PGAYGTLAELLQVS-DAYRLPVEQIAGASLFHYIVDNADTATYLADTLYRQQGGRVTFMP
AN6364.1  	EGVYGTLAELFEVN-DRYRTAVEVTAGQSLFHYVVDTDDTATKVLEILQHEKAGRVTFMP
          	 *.** **:*::*  * ** .**  ** *****:**. .*.* : : * :: .****:**

MG04988.1 	LAQLRPRKVNMPRAQNAFPLIDKLQYDEVYDKAFQQVFGKSVLCQSRDVATQYSRTHNVD
NCU07554.1	LEQLRPRQVKMPRASDAQPLISKIEFDPIYEKAFQQVFGRTIVCPNLSIASQYARTHGVD
FG06754.1 	LAQLRPKSINLPRSNDAVPLLSKISYDEAYEKAFQQVFGKVVVCPNLTVASQYARSHGVD
AN6364.1  	LNRLRTKPLNMPKASDTIPMIEKLQYDRAYEKAFQHVFGKTIICPNLQVASQYARSHGVN
          	* :**.: :::*::.:: *::.*:.:*  *:****:***: ::* .  :*:**:*:*.*:

MG04988.1 	CVTMDGDTTSKRGAVSGGYTDPTASRLLALREANKLREEYYELQEELQRLHKQIELKSQE
NCU07554.1	AITPEGDTTNKRGAMTGGFVDARRSRLEAVRRLEELRELYEQQLEDLDRIKKEDEILEQK
FG06754.1 	GITAEGDTTNKRGAMTGGYIDPRKSRLQAVQAVNKWRGEYERLLAQSRDIRQQTELKDQE
AN6364.1  	ATTPEGDRSDKRGALTGGFHDSRQSRLDAVKNLAKWRDEYETKKSRGSEIRKELEELDQL
          	  * :** :.****::**: *.  *** *::   : *  *         :::: *  .* 

MG04988.1 	TTTAVGLVRKLEQEMHKLDMEFRVKDAEFASKRKELQGLRKQLDNINVDLEKVDKEMKDM
NCU07554.1	ITSANGEERKLDQQLRQFESGFDPLKVDLRTKQLQLERERRHLEDAKMRWAAIEKNLKEF
FG06754.1 	ITAAMSELQKARERLRQAVDGFEPLRHELMNKSTHLENERGHLDAAVKRRDAVESNMNSF
AN6364.1  	ITRAVGELQKLEQQRHQVQNSSGPLRQELRSKRDLLQKQNDNLDAKRRALRNIEGNLAAL
          	 * * .  :*  :. ::          :: .*   *:  . :*:        :: ::  :

MG04988.1 	NETLSSYEAEAASEFKKALSSQEEEQLEVLGAQLGELGTQLADCSRRRLELERRKLMLEN
NCU07554.1	DENLTAYRAEMASEFKKALSAAEERQLEEFGAEEHRLQMQLKEISKKRLELEGRKKSLET
FG06754.1 	LEDLAGHETELKSDFKKNLTSAEEHQLEELGNSIQELQKEWNELSRARRDLGRQKQLLEV
AN6364.1  	KDQVDAFEAELSSPFHKALTDEEEARLESLNSNVQEYRREYQELSGKRSELETRKSVLEV
          	 : : ...:*  * *:* *:  ** :** :. .  .   :  : *  * :*  :*  ** 

MG04988.1 	ELNNNLRRREDELMSMAFDTSSGASSFAGPVGSVDEAKKELAKLKKRAAHLDSELRKAET
NCU07554.1	ELRAHLRPQEDQLRSQAFENSA-----TGGSGSFKDAQKELKKLKKAAAEVDRQLQENEE
FG06754.1 	DLRQNLQMKLDQLNSQAFENSTS----GSSAGGLKEAQRELKKAQKAQKTVEASLQEVEA
AN6364.1  	ELRENLNPRLDQLLAQDADIADE-----DGQGNIKETQREQKRLTKVLDKLAQRLAQVDE
          	:*. :*. : *:* :   : :       .  *...::::*  :  *    :   * : : 

MG04988.1 	EADRVTAQIVELENRKAHREQEQQELAREIEKMQKKLEKGLSKKAIIANKLAEFTKNIRD
NCU07554.1	KTEGIAAEIAELEAHKAQKEQEQQELQKRIDQYQKKLEKNSQTKARLLQQAAEYAKNIRD
FG06754.1 	KLDDSQARLEQLENDRAQREQAQQEISARIEKQQKRMDKSLRRKAVLTTQAAECAQTIRD
AN6364.1  	SMEQANSRVTELTQRNAESRRELEELAKSIEKHQRRMEKSMQKKAALTKQAAECAANIRD
          	. :   :.: :*   .*. .:  :*:   *:: *::::*.   ** :  : ** : .***

MG04988.1 	LGVLPEEAFDKYESMEMKS0I---EKR~LSKVNEALKKYK~HVNKKAFEQYNSFTTQQEQ
NCU07554.1	LGILPEEAFG---------~------K2LRKVNEALKKYK~HINKKAFDQYNNFTTQREQ
FG06754.1 	LGVLPEEAFDKYENMDPKT~ASFPRHG~LAKANTNMS-LG0HVNKKAFEQYNNFTTQQDQ
AN6364.1  	LGVLPDEAFTKYKNTDSNT~V---VKK~LHKVNEALKKYA~HVNKKAFEQYNNFTKQRET
          	**:**:*** . .. . .:         * *.*  :..   *:*****:***.**.*:: 

MG04988.1 	LVKRRKELDSSQQSIEDLISHLDQKKDEAIERTFKQVSREFATIFERLVPAGHGRLVIQR
NCU07554.1	LLKRRKELDTSQKSIEELIQHLDHAKDEAIERTFKQVSREFSTIFEKLVPAGHGRLVIQR
FG06754.1 	LMKRRKELDESQESIEELVEHLDRRKDEAIERTFKQVSREFTTIFGKLVPAGHGRLLIQR
AN6364.1  	LTSRREELDASQKSIDDLISVLDHRKDEAIERTFKQVSREFATIFEKLVPAGRGRLIIQR
          	* .**:*** **:**::*:. **: ****************:*** :*****:***:***

MG04988.1 	RAD-RRQTAAEESEDD--QRRGTIENYTGVGISVSFNSKEFDEQQKIQQLSGGQKS1LCA
NCU07554.1	KAAGSKNRNPEDSDEDGPSSAKGVESYSGVGISVSFNSKVMDEQQKIQQLSGGQKS1LCA
FG06754.1 	RAD-RRQEPTDESDGE---ARGAVENYTGVGISVSFNSKHLDEQQKIQQLSGGQKS1LCA
AN6364.1  	KTD-RTQRAEDDLESEDEEAKHSVENYVGVGISVSFNSK-HDDQQRIQQLSGGQKS1LCA
          	::    :   :: : :. .    :*.* ***********  *:**:********** ***

MG04988.1 	LCLIFAIQQTESSPMVVFDEVDANLDAQYRTAVASLLQSISSEAGTQFICTTFRPEIVLV
NCU07554.1	LCLIFALQAAESSPFVIFDEVDANLDAQYRTAVAALLDSISKTQKTQFICTTFRPEIVLV
FG06754.1 	LCLIFALQATESSPMVIFDEVDANLDAQYRTAVAALLDSISNEIGTQFICTTFRPEIVHV
AN6364.1  	LALVFAIQACDPAPFYLFDEIDANLDAQYRTAVAQMLKTISDSTNGQFICTTFRPEMLHV
          	*.*:**:*  :.:*: :***:************* :*.:**.    **********:: *

MG04988.1 	ADKCYGVTFTNKTSSIDCVPTEAALDFVEGQQKR-
NCU07554.1	ADKCYGVTFHNKTSTIDCVPTEDALNFVEGQVSGK
FG06754.1 	ADRCYGVTFRNKTSSIDCVSTEQALDFVEGQAKPA
AN6364.1  	AEKCYGVSFRQKASTIDVVSREEALKFVEEQKS--
          	*::****:* :*:*:** *. * **.*** * .
```
